# Whole-Genome Sequence of “Candidatus Liberibacter asiaticus” from a Huanglongbing-Affected Citrus Tree in Central Florida

**DOI:** 10.1128/genomeA.00169-15

**Published:** 2015-03-19

**Authors:** Z. Zheng, X. Sun, X. Deng, J. Chen

**Affiliations:** a Department of Plant Pathology, South China Agricultural University, Guangzhou, Guangdong, China; b Florida Department of Agriculture and Consumer Services, Division of Plant Industry, Gainesville, Florida, USA; c San Joaquín Valley Agricultural Sciences Center, USDA-ARS, Parlier, California, USA

## Abstract

Here, we report the draft genome sequence of “Candidatus Liberibacter asiaticus” strain FL17, isolated from a huanglongbing (HLB)-affected citrus tree in central Florida. The FL17 genome comprised 1,227,253 bp, with a G+C content of 36.5%, 1,175 predicted open reading frames, and 53 RNA genes.

## GENOME ANNOUNCEMENT

“Candidatus Liberibacter asiaticus” is an unculturable alphaproteobacterium associated with citrus huanglongbing (HLB) (yellow shoot disease, also called greening disease), a devastating citrus disease worldwide (1–3). In the United States, “*Ca*. Liberibacter asiaticus” was first discovered in Florida in 2005 (4). HLB is now found in all citrus-growing regions in Florida. Due to the lack of *in vitro* culture, the characterization of “*Ca*. Liberibacter asiaticus” has mainly relied on DNA sequence analyses. A whole-genome sequence of “*Ca*. Liberibacter asiaticus” (strain Psy62) was first obtained through a metagenomics approach from an infected Asian citrus psyllid in 2009 (5), followed by sequences of multiple strains from different geographical locations (6–9). Here, we report a draft whole-genome sequence of a “*Ca*. Liberibacter asiaticus” strain directly from an HLB-affected citrus tree in central Florida.

“*Ca*. Liberibacter asiaticus” strain FL17 was originally collected from a citrus tree showing typical HLB symptoms (yellowing and mottling) in a nursery in Polk County, FL. Total DNA from an infected leaf petiole was extracted and used for whole-genome sequencing, according to a previously developed procedure (6, 7). Briefly, bacterial DNA was enriched using a NEBNext microbiome DNA enrichment kit (New England BioLabs, Inc., Ipswich, MA) and further amplified using the REPLI-g minikit (Qiagen, Inc., Valencia, CA). Sequencing was performed on an Illumina MiSeq platform (Illumina Inc., San Diego, CA).

MiSeq generated a total of 5.17 × 10^7^ reads (mean, 301 bp) or a total of 1.56 × 10^10^ bp of data. Using the whole-genome sequences of “*Ca*. Liberibacter asiaticus” strain Psy62 (5) and two “*Ca*. Liberibacter asiaticus” phages/prophages (SC1 and SC2) (10) as references, a total of 213,418 reads were identified by using the stand-alone BLAST software (11). The sequence reads were collected by using a Perl script. Assembly was carried out by Velvet 1.2.0 (https://www.ebi.ac.uk/~zerbino/velvet/) (12) and the CLC Genomics Workbench 7.5 software, resulting in 3 contigs ranging from 13,921 bp to 1,198,110 bp, with ~50× coverage. The draft FL17 genome sequence comprised 1,227,253 bp, with a G+C content of 36.5%. Annotation through the RAST server (http://rast.nmpdr.org) (13) identified 1,175 open reading frames (ORFs) and 53 RNA genes.

The citrus-origin FL17 strain genome had a >99% coverage of the psyllid-origin Psy62 strain genome (1,227,204 bp) (5), and both include an SC1 prophage. However, strain Psy62 was later confirmed to harbor an SC2-like prophage sequence, named FP2 (14). In contrast, other than the genes shared by SC1 and SC2, strain FL17 did not have SC2-specific prophage sequences. Therefore, strain FL17 does not appear have an SC2-like prophage.

### Nucleotide sequence accession numbers.

This whole-genome shotgun project has been deposited at DDBJ/EMBL/GenBank under the accession no. JWHA00000000. The version described in this paper is version JWHA01000000.
